# An improved clear cell renal cell carcinoma stage prediction model based on gene sets

**DOI:** 10.1186/s12859-020-03543-0

**Published:** 2020-06-08

**Authors:** Fangjun Li, Mu Yang, Yunhe Li, Mingqiang Zhang, Wenjuan Wang, Dongfeng Yuan, Dongqi Tang

**Affiliations:** 1 School of Information Science and Engineering, Shandong University, supported by Shandong Provincial Key Laboratory of Wireless Communication Technologies, Jinan, 250100 China; 2 Center for Gene and Immunothererapy, The Second Hospital of Shandong University, Jinan, 250033 China

**Keywords:** Feature selection, Machine learning, Clear cell renal cell carcinoma, Cancer stage

## Abstract

**Background:**

Clear cell renal cell carcinoma (ccRCC) is the most common subtype of renal cell carcinoma and accounts for cancer-related deaths. Survival rates are very low when the tumor is discovered in the late-stage. Thus, developing an efficient strategy to stratify patients by the stage of the cancer and inner mechanisms that drive the development and progression of cancers is critical in early prevention and treatment.

**Results:**

In this study, we developed new strategies to extract important gene features and trained machine learning-based classifiers to predict stages of ccRCC samples. The novelty of our approach is that (i) We improved the feature preprocessing procedure by binning and coding, and increased the stability of data and robustness of the classification model. (ii) We proposed a joint gene selection algorithm by combining the Fast-Correlation-Based Filter (FCBF) search with the information value, the linear correlation coefficient, and variance inflation factor, and removed irrelevant/redundant features. Then the logistic regression-based feature selection method was used to determine influencing factors. (iii) Classification models were developed using machine learning algorithms. This method is evaluated on RNA expression value of clear cell renal cell carcinoma derived from The Cancer Genome Atlas (TCGA). The results showed that the result on the testing set (accuracy of 81.15% and AUC 0.86) outperformed state-of-the-art models (accuracy of 72.64% and AUC 0.81) and a gene set FJL-set was developed, which contained 23 genes, far less than 64. Furthermore, a gene function analysis was used to explore molecular mechanisms that might affect cancer development.

**Conclusions:**

The results suggested that our model can extract more prognostic information, and is worthy of further investigation and validation in order to understand the progression mechanism.

## Introduction

Clear cell renal cell carcinoma (ccRCC) accounts for 60–85% of RCC [1, 2], which represents 2–3% of all cancers with a general annual increase of 5% [3, 4]. ccRCC is usually asymptomatic in the early stages, with about 25–30% of patients having metastasis by the time of diagnosis [5]. Moreover, patients who had localized ccRCCs removed by nephrectomy have a high risk of metastatic relapse [6]. ccRCC has high resistance to chemotherapy and radiotherapy, leading to poor prognosis [7, 8]. Detecting ccRCC in the early stage can help prevent and treat cancer at early stages. Also, understanding key genetic drivers for progression can help to develop new treatments.

Gene expression profiling has the potential for the classification of different tumor types since they play an important role in tumor development and metastasis. Machine learning-based methods which make use of gene expression profiling have been developed for discriminating stages in various cancers [9], including ccRCC [10, 11]. Rahimi [9] recommended using a multiple kernel learning (MKL) formulation on pathways/gene sets to learn an early- and late-stage cancer classification model. Jagga [10] and Bhalla [11] trained different machine learning models using genes selected by Weka and achieved a maximum AUROC of 0.8 and 0.81 on ccRCC respectively. Although some researchers have distinguished early and advanced stages of ccRCC using the classification models, the stability of the classification model is not guaranteed and there is still room for improvement in model performance.

This work aimed to extract significant features from high-dimensional gene data using data mining techniques and make more accurate and reliable predictions of ccRCC tumor stages with machine learning algorithms. For data preprocessing, we used the Chi-merge binning and WOE encoding algorithm to accomplish data discretization, thus reducing the impact of statistical noise and increasing the stability of the classification model. For gene selection, a joint selection strategy to remove irrelevant/redundant features was proposed, and the final FJL-set with 23 genes was derived as an aggregated result. Specifically, we aggregate Fast-Correlation-Based Filter search (FCBFSearch), joint statistical measures (the information value, the linear correlation coefficient, and variance inflation factor) and logistic regression-based feature selection. For the classification model, five different supervised machine learning algorithms were evaluated on an independent testing set. Finally, a simple and comprehensible SVM based prediction model using 23 selected genes performed best with an accuracy of 81.15% and AUC 0.86 — higher than the state-of-the-art method with fewer genes.

## Materials

The RNAseq expression data along with their clinical information for Kidney Renal Clear Cell Carcinoma (KIRC) samples from The Cancer Genome Atlas (TCGA) project were used to distinguish between early- and late-stage ccRCC. RSEM values of KIRC used as gene expression values and clinical annotations for cancer patients were derived from UCSC Xena (https://xenabrowser.net/datapages/). FPKM values of KIRC were derived in TCGA for comparison with RSEM.

Samples with Stage I and II were considered as early-stage (i.e. localized cancers) and the remaining samples with Stage III and IV were labeled as late-stage cancers. After this processing, 604 samples from early- and late- stages were retained. 80% samples (482 samples) were picked randomly as the training set and the remaining 20% (122 samples) were used as the independent test set. Table 1 shows the datasets used in this study.

**Table 1:** Summary of TCGA - KIRC that was used in the training and test set

Stage		Sample Number		Training set		Testing set	
Early	Stage I	361	293	288	234	73	59
	Stage II		68		54		14
Late	Stage III	243	139	194	111	49	28
	Stage IV		104		83		21

## Methods

**Fig. 1:**
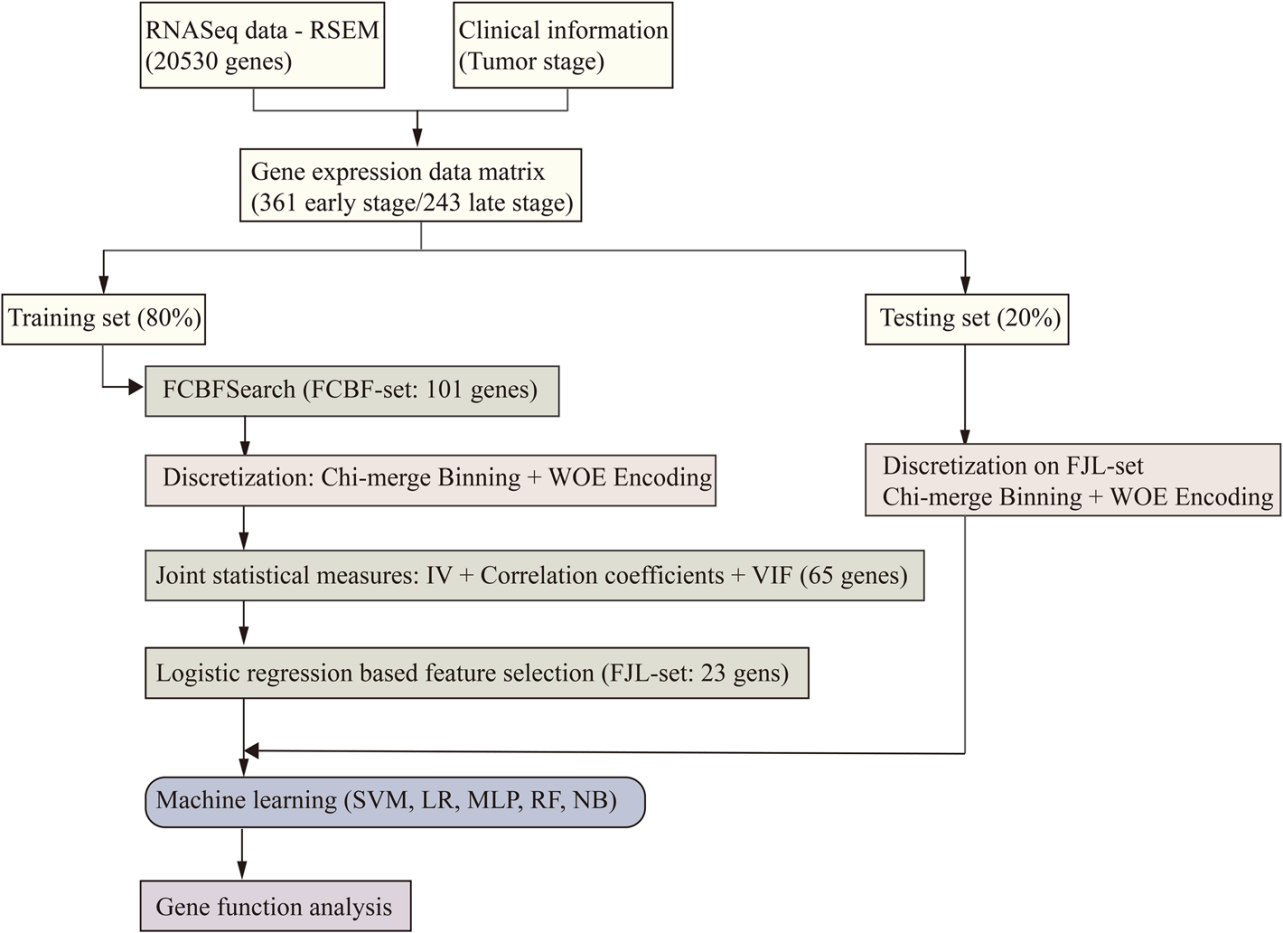
The overall algorithm framework

Feature selection and classification algorithms with preprocessed gene expression profiles were used to detect early- and late-stage samples. Due to the wide range and highly correlated nature of gene expression data, the performance of classification models with raw features were not robust. Therefore, feature selection was conducted before classification, and only on the training set. Five supervised machine learning algorithms were used on gene sets to predict their pathological stages. Figure 1 demonstrates the overall algorithm framework used in this work.

### Feature preprocessing

To increase the stability and robustness of the classification model, Chi-merge binning and WOE encoding for discretizing genetic features were conducted. The range of each numeric RSEM attribute for different genes can be very wide. While some extremely large values seldom appear, they can cause prediction impairment because of seldom reversal patterns and extreme values. Grouping similar properties with similar predictive intensity will increase the instability of models and allow the understanding of the logical trend of “early−/ late-stage” bias of each feature.

#### Discretization

##### Chi-merge binning

Binning and encoding are techniques purposed to reduce the impact of statistical noise. It is widely used in credit risk prediction and other applications. However, no prior works apply this method to cancer classification problems. Instead, they put the normalized genetic features into machine learning models directly.

Chi-merge is the most widely used automatic grading algorithm. It is partitioned in such a way that the early-stage and late-stage samples are as different as possible in the proportion of adjacent boxes. The disadvantage of Chi-merge is that it requires mass computation, so it may not be a good choice for selecting features from all genes.

##### WOE encoding

After binning, the original numeric characteristics are transformed into categorical ones, and it is impossible to put the discretized variables directly into the model. Therefore, variables of discrete type need to be coded. WOE encoding was used in our experiments to encode these categorical variables.

Weight of evidence (WOE) is based on the ratio of early-stage to late-stage samples at each level. It weighs the strength of feature attributes to distinguish between early- and late-stage accounts.

1 $$ {WOE}_i=1\mathrm{n}\left(\frac{E_i/E}{L_i/L}\right)=1\mathrm{n}\left(\frac{E_i/{L}_i}{E/L}\right)=1\mathrm{n}\left(\frac{E_i}{L_i}\right)-1\mathrm{n}\left(\frac{E}{\;L}\right) $$

Here *E*_*i*_ is the number of early-stage samples in bin *i*, *L*_*i*_ is the number of bad early-stage samples in bin *i*, *E* is the total number of early-stage samples, and *L* is the total number of bad early-stage samples.

#### Standardization

In the second set of experiments, the RSEM values were transformed using log2 after adding 1.0. Then the log2 transformed values were normalized. The following equations were used for computing the transformation and normalization:

2 $$ x={\log}_2\left( RSEM+1\right) $$

3 $$ z=\frac{x-\overline{x}}{s} $$

Where *x* is the log-transformed gene expression, $\overline{x}$ is the mean of training samples, and s is the standard deviation of the training samples.

### Feature selection

A hybrid feature selection method was developed which aimed to produce a feature subset from aggregated feature selection algorithms. All these algorithms were conducted on the training set. The feature selection method was composed of three parts: (1) FCBFSearch, (2) joint statistical measures, and (3) logistic regression-based feature selection. In this way, irrelevant/redundant attributes in data sets can be removed, the instability and perturbation issues of single feature selection algorithms can be alleviated, and the subsequent learning task can be enhanced.

#### Fast correlation-based filter search

When there are a lot of variables, there is a strong relevance/redundance between the variables. If all the variables are put together into classification models, the significance of important variables is reduced, and in extreme cases, sign distortion occurs. The Fast Correlation-Based Filter (FCBF) Search algorithm is a feature selection algorithm based on information theory [12], which takes into account both feature correlation and feature redundancy. It uses dominant correlation to distinguish related features in high-dimensional datasets.

FCBFSearch was performed on the original training data without data preprocessing. In addition, a random sampling method was used to select the robust features. FCBFSearch was conducted 10 times with random sampling 10-fold cross-validation every time on the training dataset, after which 10 subsets of features were obtained. The features with an overlap number of more than 8 were selected for the data preprocessing and the following joint statistical measures processions.

#### Joint statistical measures

Joint statistical feature selection was done on preprocessed FCBFSearch features. The method combines various statistical measures to assess feature importance and relevance and filter out redundant features.

1. Univariate Analysis

The information value (IV) is used to assess the overall predictive power of the feature, i.e. the ability of the feature to separate early-and late-stage samples. It expresses the amount of information of the predictor in separating early- from late-stage in the target variable.

$$ IV=\sum \left(\frac{G_i}{G}-\frac{B_i}{B}\right)\ln \left(\frac{G_i/G\ }{B_i/B\ }\right) $$$$ \mathrm{IV}=\sum \left(\frac{{\mathrm{G}}_{\mathrm{i}}}{\mathrm{G}}-\frac{{\mathrm{B}}_{\mathrm{i}}}{\mathrm{B}}\right)\ln \left(\frac{{\mathrm{G}}_{\mathrm{i}}/\mathrm{G}}{{\mathrm{B}}_{\mathrm{i}}/\mathrm{B}}\right) $$ (4).

Where *G*_*i*_ is the proportion of early-stage samples of bin *i* in all early-stage samples and *B*_*i*_ is the proportion of late-stage samples of bin *i* in all late-stage samples.

IV < 0.02 represents an unpredicted variable, 0.02–0.10 is weakly predictive, 0.10–0.30 is moderately predictive, and > 0.30 is strongly predictive. In the experiment, we rejected variables whose IV was lower than 0.1.

(2) Multivariate Analysis

The linear correlation coefficient was used to measure the correlation between two variables. The larger the absolute value of the linear correlation coefficient is, the more likely it is to be a linear expression for another variable. Linear correlation has two meanings: positive correlation and negative correlation. It is desirable to avoid both of these situations because it is hoped that the correlation between the two variables is as small as possible. In the present study, 0.7 was chosen as the baseline. If the absolute value of the correlation coefficient was greater than 0.7, the one with lower IV score was selected.

After this, collinearity analysis was performed since the collinearity problem tends to reduce the significance of a variable. The Variance Inflation Factor (VIF) was used to evaluate multivariate linear correlation.

5 $$ {VIF}_i=\frac{1}{1-{R}_i^2} $$

Where *R*_*i*_ is the *R*^2^ value of *x*_*i*_ and {*x*_1_, *x*_2_, …, *x*_*i* − 1_, *x*_*i* + 1_, *x*_*i* + 2_, …, *x*_*N*_} . When the calculated VIF is far less than 10, there is no collinearity problem.

#### Logistic regression-based feature selection

In the present study, logistic regression (LR) was used as the classification model in feature selection progress in order to find which factors were influential in discriminating early- and late-stage samples, and how these factors quantitatively affect the model.

To guarantee the validity and significance of the variables sent to the logistic regression model, we checked the coefficients and *p* values of the input variables which indicate the influence of the independent variable on the dependent variable and whether early- and late-stage genetic expression significantly change. Some variables’ p values are higher than 0.1 before checking, and it means that there is no obvious correlation between the two parameters. In our study, we filtered variables whose *p*-value exceeded the threshold 0.1 and the values of coefficients were positive.

### Classification algorithm

Five machine learning algorithms: Support Vector Machine (SVM), Logistic Regression, Multi-Layer Perception (MLP), Random Forest (RF) and Naive Bayes (NB) were used for generating the classification models. RBF kernel of SVM at different parameters, gamma∈[10^− 9^, 10^− 7^, ..., 10, 10^3^], c∈[− 5, − 3, ..., 13, 15] was used for optimizing the SVM performance. SVM, MLP, RF, and NB were implemented using the Sklearn package in Python.

#### 10-fold cross-validation

The five supervised machine learning algorithms were trained on the subset features from feature selection and further validated by 10-fold cross-validation.

#### Independent dataset test

An independent testing set is used to exclude the “memory” effect or bias for trained classification models. We did not use this testing set for feature selection or model training. We only evaluated the performance of the classification model on it, and the model was trained on the training set.

### Analysis of selected genes

The Database for Annotation, Visualization and Integrated Discovery (DAVID, version 6.7) [13] and KEGG [14] database was used to explain the meaning of functional from the molecular or higher levels and associate the genes with related pathways. As a main bioinformatics database for analyzing gene function and understanding the biological functions, GO is integrated with other databases in DAVID [15]. A meaningful biological explanation for the selected genes through the enrichment analysis, and correlating genes with diseases in the mechanism is needed. *P* < 0.05 was considered statistically significant.

## Results

Experiments were performed on the TCGA - KIRC dataset that was constructed with labeling strategies shown in Table 1. The results of every feature selection procedures and performance of the classification algorithm are shown.

### Experiment settings

The feature selection process and classification models were conducted on the training set while the performance of models was evaluated using 10-fold cross-validation on the training set as well as on the independent testing set. We implemented the initial FCBFSearch in Weka 3.8, and the attribute evaluator ‘SymmetricalUncertAttributeSetEval’ with the search method of ‘FCBFSearch’ was used to accomplish this process. All data preprocessing feature extraction, joint statistical feature selection measures, and classification algorithms were in Python programming language, and the related code is publicly available in the github (https://github.com/lfj95/FJL-model). The details of experimental settings in compared methods are described in the Supplementary Methods.

### Data preprocessing results

#### Binning and encoding deals with the long tail data distribution

**Fig. 2:**
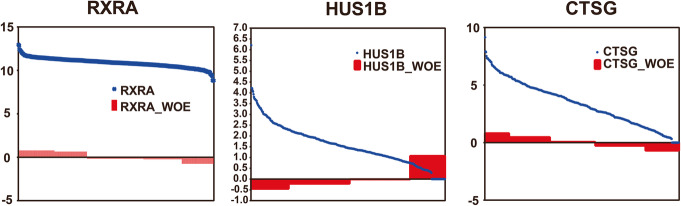
Comparison of data distribution of 3 representative genes before and after binning and encoding

To show the role of binning and encoding, the data distribution of 3 representative genes were plotted. Expression values of these 3 genes (Fig. 2) shows that the original dataset had long tail distributions, and the probability of occurrence of maximum value was very small. In addition, this kind of data distribution can cause great interference to the classification procedure so that it is unstable. After Chi-merge binning and WOE encoding, the training data were discretized and mapped to values between − 3 and 3. These results indicate that binning and encoding could normalize variables to similar scales and reduce the effect of the data distribution.

### Feature selection results

In this section, the results of each feature selection step: (1) FCBFSearch, (2) joint statistical measures, and (3) logistic regression-based feature selection are shown.

#### FCBFSearch

The selection frequencies of genes selected by FCBFSearch are shown in Table S2. The 101 genes that were selected more than 8 times are marked in bold. FCBFSearch was conducted on gene data without preprocessing, following the discretization process which eliminated 6 genes whose maximum bin occupied more than 90% during the preprocessing process. So only 95 genes went to joint statistical measures.

#### Joint statistical measures

**Fig. 3:**
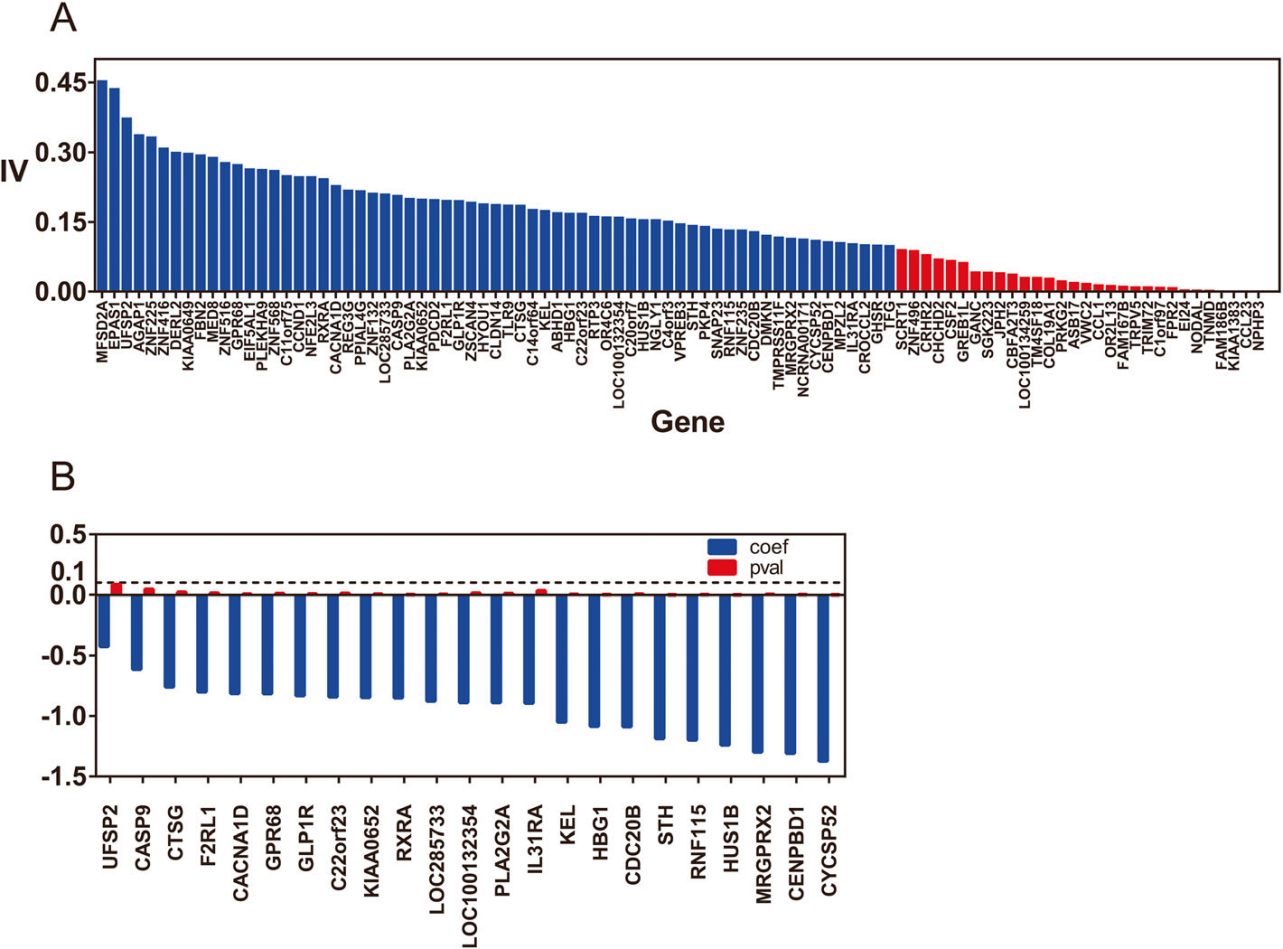
Performance of feature selection algorithms. (**a**) IV score of 95 genes (higher than 0.1 in blue, lower than 0.1 in red). (**b**) Validity and significance test of variables. The coefficients of all selected variables are negative but the *p* values of some genes are higher than 0.1. After the phase-out, the significance of residual variables are guaranteed

The information value was employed for finding the importance of genes, linear correlation coefficient, and the variance inflation factor for discovering associations among genes. Thirty genes whose IV score was lower than 0.1 were removed (Table S3) since the predictor was not useful for modeling. After this process, there were 65 genes left, and gene MFSD2A had the highest IV 0.455. In addition, 27 genes reached an IV score of 0.2, as shown in Fig. 3A. Therefore, the prediction ability of individual variables collected was strong, and the prediction ability of selecting the appropriate feature combination was available.

Correlation coefficients between genes were all lower than the threshold value 0.7 and the calculated VIF were all far less than 10. So, no genes were removed in this step, indicating that genes included in the classification model all had high importance and low correlation.

#### Logistic regression-based feature selection

To guarantee the correctness and significance of the variables sent to the logistic regression model, the coefficients and *p* values of the input variables were checked to eliminate variables that were not valid and not significant, respectively. Figure 3B shows variables before and after filtering, the coefficients and p values which indicate the influence of the independent variable on the dependent variable and whether early- and late-stage genetic expression significantly changed. As can be seen, some variables’ p values were higher than 0.1 before checking. This means that there is no obvious correlation between the two parameters. The variable size was reduced from 65 to 23 after stepwise iteration removed insignificant variables, while the remaining *p*-values did not exceed the threshold 0.1 and the values of coefficients were all negative.

### Classification results

In this section, the classification results of the model and the baseline models are shown. Prediction models on the independent test set with 122 samples, in terms of area under the receiver operating characteristic curve (AUC), accuracy, Matthews Correlation Coefficient (MCC), specificity, and sensitivity were evaluated. The generalization ability of the algorithm was also reflected by a 10-fold cross-validation experiment. For each fold, separate classifiers were trained, and the result finally obtained was the average of 10-folds.

#### FJL-set-based models

Twenty-three genes in the FJL-set with the preprocessing method shown in 3.1.1 were used to classify “early- and late-stage” on the five machine learning algorithms -- SVM, MLP, Random Forest, Decision Tree, and Naive Bayes (Table 2).

**Table 2:** The performance of machine learning based-models developed using FLJ-set of 23 selected features on the training set with 10-fold cross-validation set and independent testing set for gene data without discretization

Algorithms	Methods	Performance Measures on test set				
		Sensitivity	Specificity	Accuracy(%)	MCC	AUC
Logistic Regression	10-fold	0.750	0.805	78.45	0.556	0.855
	Testing	0.756	0.767	77.87	0.554	**0.860**
SVM	10-fold	0.680	0.868	79.27	0.562	0.852
	Testing	0.714	0.877	81.15	0.603	**0.860**
MLP	10-fold	0.706	0.828	77.83	0.508	0.840
	Testing	0.776	0.836	81.15	0.609	0.858
Naive Bayes	10-fold	0.695	0.820	77.17	0.519	0.828
	Testing	0.735	0.836	79.51	0.572	0.819
Random Forest	10-fold	0.499	0.866	71.75	0.398	0.764
	Testing	0.612	0.863	76.23	0.496	0.828

Sensitivities of all the models were in the range of 0.612–0.776 with the highest sensitivity of 0.776 for MLP. Specificities of the models varied in a range with the lowest of 0.767 for logistic regression and the highest of 0.877 for SVM. The best sensitivity-specificity trade-off was observed for the SVM Classifier with a sensitivity of 0.714 and specificity of 0.877. The classification accuracy of the generated prediction models ranged from 76.23% for Random Forest to 81.15% for SVM, and the AUC score ranged from 0.819 for Naive Bayes to 0.860 for SVM. Based on accuracy and AUC, we inferred the SVM based prediction model outperformed the other four machine learning algorithms implemented in the study. The MCC of the models developed in the study was between 0.496 and 0.609. It is notable that among the four evaluated prediction models, the model based on SVM had the highest specificity, accuracy, AUC.

**Fig. 4:**
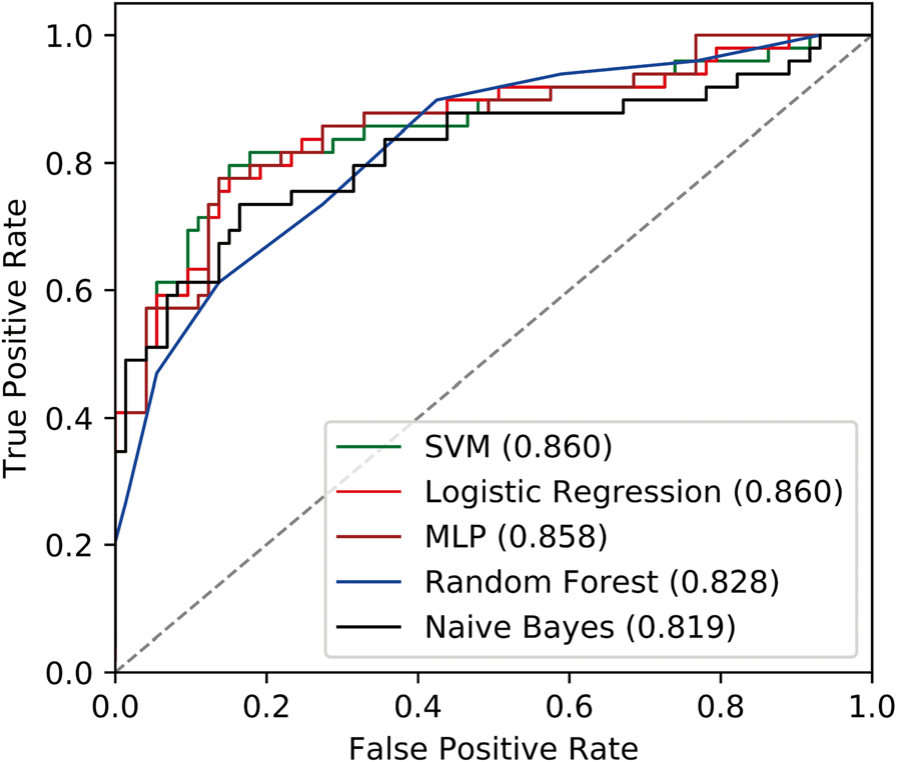
Receivers Operating Characteristic curve (ROC) for all the five classifiers with discretization

The ROC curve (Fig. 4) was plotted to summarize the performance of different models in discriminating early- and late-stage ccRCC in the preprocessed test data sets. One hundred and twenty-two test samples were used to evaluate the prediction power of the five classifiers with two preprocessing methods. Among the prediction models, SVM and Logistic Regression achieved the maximum value of 0.860 for AUC. Naive Bayes had the least AUC of 0.819, about 0.04 lower than SVM. In real-word applications, logistic regression is also a good choice.

#### No feature selection based models

We first conducted experiments without feature selection to explain the performance of models developed using machine learning techniques. We used 20,530 gene features with the preprocessing method as shown in 3.1.2. The classification result on the testing set is shown in Table 3.

**Table 3:** The performance of machine learning-based models developed using different sets of selected features, which include whole gene sets without feature selection, RCSP-set-Weka-Hall, FCBF-set, and FJL-set

Features	Algorithms	Methods	Performance Measures				
			Sensitivity	Specificity	Accuracy(%)	MCC	AUC
Whole gene set (20,530 genes)	SVM	10-fold	0.182	0.943	63.25	0.198	0.709
		Testing	0.020	1.000	60.66	0.111	0.806
	LR	10-fold	0.590	0.777	69.91	0.370	0.683
		Testing	0.673	0.863	78.69	0.551	0.768
RCSP-set-Weka-Hall (38 genes)	SVM	10-fold	0.696	0.697	70.35	0.386	0.769
		Testing	0.735	0.808	77.87	0.541	0.844
FCBF set (101 genes)	SVM	10-fold	0.727	0.758	74.23	0.475	0.793
		Testing	0.776	0.740	75.41	0.506	0.826
	LR	10-fold	0.678	0.742	71.57	0.415	0.768
		Testing	0.612	0.808	72.95	0.429	0.789
FJL set (23 genes)	Discretization +SVM	10-fold	0.680	0.868	79.27	0.562	0.852
		Testing	0.714	0.877	81.15	0.603	**0.860**
	Discretization + LR	10-fold	0.750	0.805	78.45	0.556	0.855
		Testing	0.756	0.767	77.87	0.554	**0.860**
	Discretization +SVM	100 random test sets	0.710	0.788	75.64	0.496	0.831
	Discretization + LR	100 random test sets	0.647	0.876	78.32	0.542	0.842

The performance of AUC on the testing set was 0.806 in SVM and 0.768 in LR. The results of traditional machine learning algorithms before feature selection were not high, especially for logistic regression, whose performance was highly affected by the wide range and highly correlated gene expression data. Therefore, feature selection is essential to improve prediction accuracy.

#### RCSP-set-Weka-hall based models

The best results were compared with Bhalla’s results. The research [11] that Bhalla et al. did selected a subset of genes that are components of cancer hallmark processes and obtained a good performance of the model. We conducted experiments with these 38 genes on both training set with 10-fold cross-validation and on a test set. The preprocessing method used is as described in 3.1.2, the same as that used in their study. The classification result on the testing set is shown in Table 3.

As reported in their paper, they achieved an accuracy of 77.7% with AUC 0.83 on their training data and accuracy of 72.64% with AUC of 0.78 on their validation data with 104 test samples. In the present experiment, their method was repeated in Python and an accuracy of 77.87% with AUC of 0.844 with SVM on our test data with 122 test samples was obtained, while the results on the training set using 10-fold cross-validation were 70.35% in accuracy and 0.769 in AUC (Table 3).

#### FCBF-set-based models

In this section, the feature selection was performed by Weka on preprocessed data with the method described in 3.1.2 and the number of features was reduced from 20,530 to 101 features (FCBF-set). LR based models did not perform well with these 101 genes, with an accuracy of 72.95% and AUC of 0.789 on the test set. SVM based models gave the best performance with an accuracy of 74.23% with AUC 0.793 on the training data using 10-fold cross-validation and an accuracy of 75.41% with AUC of 0.826 on the testing set (Table 3), which were higher than the results of RCSP-set-Weka-Hall based model. For certainty of results, we made 100 random sets from 60% validation samples to test the biomarkers in these random sets as well, and the mean of randomized experiments is shown in Table 3.

It can be seen that FJL set-based models perform best, which confirms that the genes selected with our method have a certain significance for the division of pathological stages. Also, there is a consistency between the results of 10-fold cross-validation and results on the testing set.

Besides, FPKM values were experimented in the same process with RSEM. Accuracy and AUC are also better than RCSP-set-Weka-Hall set, as were shown in the Table S5, indicating that the experimental method is also applicable to FPKM and it also can get a good classification result.

### Biological mechanisms identified by selected genes

Many filtered genes in our method were confirmed to associate with tumor in the previous literature. UFSP2 combined with the nuclear receptor coactivator ASC1 is involved in the development of breast cancer [16]. GPR68 is a mediator interacting with pancreatic cancer-associated fibroblasts and tumor cells [17]. RXRA mutation drives about a quarter of bladder cancer [18]. CACNA1D mutation causes increased Ca^2+^ influx, further stimulating aldosterone production and cell proliferation in adrenal glomerulosa [19]. CASP9 expression has an apoptosis-inducing and anti-proliferative effect in breast cancer [20]. High expression of PLA2G2A can cause short survival in human rectal cancer [21]. KIAA0652 (ATG13) mediates the inhibition of autophagy in DNA damage via the mTOR pathway [22]. CTSG (Cathepsin G) is thought to be an effective therapeutic target in acute myeloid leukemia patients [23] and could rapidly enhance NK cytotoxicity [24]. HUS1b is confirmed to have the function of checkpoint activation in the response to DNA damage, and its overexpression induces cell death [25]. Saitohin polymorphism is associated with the susceptibility of late-onset Alzheimer’s disease [26] and does not associate with the cancer. RNF115 is broadly overexpressed in ERα-positive breast tumors [27]. Wintergerst L et al. [28] reported that CENPBD1 can predict clinical outcomes of head and neck squamous cell carcinoma patients. Tumor cells produce IL-31, and IL-31 and its receptor are confirmed to affect the tumor microenvironment [28].

**Fig. 5:**
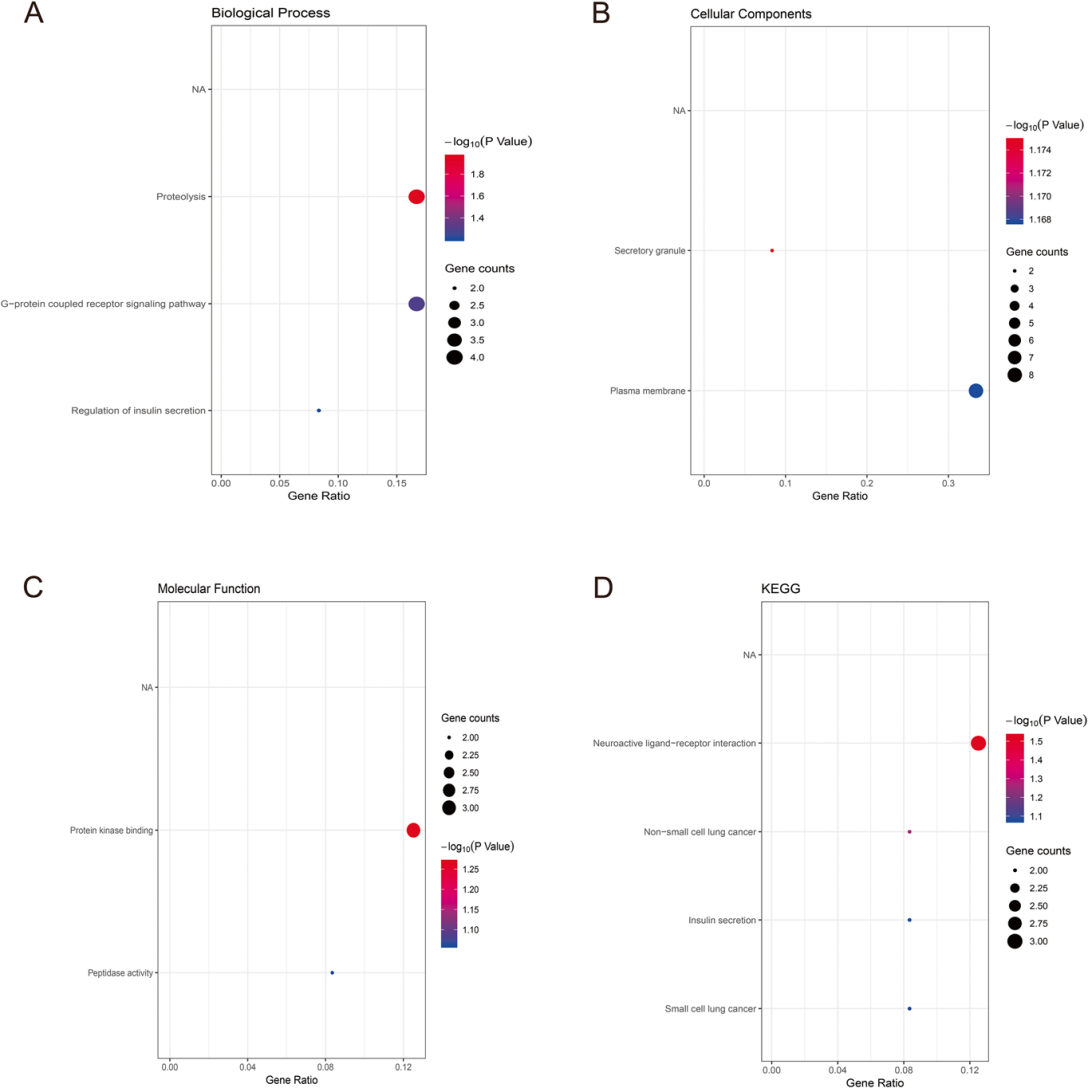
GO and KEGG pathway enrichment analysis of selected genes

Functional roles of the 23 hub genes are shown in Table S4. The results in GO analysis showed that the biological processes (BP) were proteolysis, G-protein coupled receptor signaling pathway, and regulation of insulin secretion (Fig. 5). G-protein coupled receptor signaling mediates kidney dysfunction [29]. Also, elevated circulating levels of urea in chronic kidney disease can cause the dysfunction of secretory insulin [30]. Genetic changes in molecular function (MF) show that there are enrichment terms including protein kinase binding and peptidase activity. The most varied term in cell component (CC) was the extracellular region. KEGG analysis found that the selected genes were mostly enriched in the Neuroactive ligand-receptor interaction.

## Discussion

In this study, we presented an effective computational framework with a higher capability to discriminate the stage of ccRCC tumor samples. Previous work identified a panel with these genes that can use gene expression data to effectively distinguish between early and late ccRCC patients [11]. Different machine learning algorithms have also been applied [9, 11]. However, given the selected gene set, we speculated that the prediction performance can be improved with better feature processing methods. The major contributions of the proposed method are (1) an improved feature preprocessing method by discretization of gene expression data through Chi-merge binning and WOE encoding, (2) gene panel selection through FCBFSearch, joint statistical measures (IV, the linear correlation coefficient and VIF), and logistic regression-based feature selection. We eliminated noisy and extraneous genetic features during this process and finally obtained a hub gene set (FJL-set) which consists of 23 genes, (3) validation of the performances of machine learning algorithms. Our model can achieve a higher predictive accuracy than baseline models while using less selected genes, and (4) analyzation of the genes’ functions. It was found that the targeted genes were confirmed to associate with cancer in the existing research.

There are two main directions of our future work. We will first try other basic feature selection methods other than FCBFSearch on the whole gene set, leading to more accurate classifiers. Then this discrimination algorithm will be applied to other diseases and datasets. By doing so, we will be able to validate the generalization ability of our model.

## Supplementary information

**Additional file 1: Table S1.** The differences of experimental settings between the compared method in the reference and in this article. **Table S2.** Gene selection result of FCBFSearch with 10 times of 10-fold cross validation in training set. **Table S3.** Gene selection result of joint statistical measures, following 30 genes were removed during this process. **Table S4.** Functional roles of 23 hub genes with selected times ≥8. **Table S5**. The performance of machine learning-based models using the value of FPKM and RSEM respectively.

## Data Availability

All code are available at https://github.com/lfj95/FJL-model.
