# Supplementary material for: An improved clear cell renal cell carcinoma stage prediction model based on gene sets
Source: BMC Bioinformatics. 2020 Jun 8;21:232. doi: 10.1186/s12859-020-03543-0 (PMC7278205; doi:10.1186/s12859-020-03543-0)
Supplement: Supplementary file 1 — Additional file 1: Table S1. The differences of experimental settings between the compared method in the reference and in this article. Table S2. Gene selection result of FCBFSearch with 10 times of 10-fold cross validation in training set. Table S3. Gene selection result of joint statistical measures, following 30 genes were removed during this process. Table S4. Functional roles of 23 hub genes with selected times ≥8. Table S5. The performance of machine learning-based models using the value of FPKM and RSEM respectively. [file 12859_2020_3543_MOESM1_ESM.docx]

**Supplementary Information File**

**Methods**

1. The details of experimental settings in compared methods

1.1 The data preprocessing process

In the compared method, caret package in R was used for normalizing the data. While in our experiment, we used package scikit-learn [1] in python. (Table S5)

1.2 The feature selection process

In our experiment, the feature selection process is composed of three parts, and the first part ‘FCBFSearch’ is similar to the ‘Weka-Based’ method in the compared experiment, with software package Weka [2]. (Table S5) There were only little differences in methods of selecting the robust features. In the compared method, “In order to select the robust features, the train data set was split into the ratio of 80:20 for 100 times followed by features selection using Weka every time on the training dataset. From this resampling process, we obtained 100 sub-sets of features. The feature sub-set depicting maximum overlap in terms of number of features with other sub-sets was selected for model development.” [3]

Apart from this, all congifurations were the same, with attribute evaluator named ‘SymmetricalUncertAttributeSetEval’ and search method of ‘FCBFSearch’ in weka to accomplish this process.

1.3 Classification models

In our experiment, classification models were implemented with package scikit-learn [1] in python, while in the compared method, “we used two sofware packages SVM^light^  and Weka. Random forests, SMO, Naïve Bayes, J48 were implemented using Weka sofware.” (Table S1) [3]

**Table S1** The differences of experimental settings between the compared method in the reference and in this article.

| Experimental Settings | | In the reference | In this article |
| --- | --- | --- | --- |
| Data Preprocessing | Implementation Tools | caret package in R | sklearn.preprocessing in python |
| Feature Selection | Resampling Process | the train data set was split into the ratio of 80:20 for 100 times | the train data set was split into 10-fold for 10 times |
|  | Feature Sub-set | maximum overlap in terms of number of features | Genes been selected for more than 8 times |
| Classification Models | Implementation Tools | packages SVM^light^ and Weka | package sklearn in python |

**Table S2.** Gene selection result of FCBFSearch with 10 times of 10-fold cross validation in training set.

| Times | Gene |
| --- | --- |
| 10 | **ZNF568, PKP4, EPAS1, MRAP, ZSCAN4, OR1K1, ZNF132, GPR68, NODAL, PPIAL4G, FBN2, KIAA0649, TNMD, GHSR, LOC100132354, OR4C6, CACNA1D, ABHD1, PLA2G2A, ZNF496, TRPV5, MPZL2, RXRA, FAM167B, PDCD2, CHCHD2, C1orf97, C11orf75, CRHR2, LOC285733, MRGPRX2, CBFA2T3, KCNK18, OLIG3, CASP9, STH, JPH2, KIAA0652, HUS1B, CYCSP52, RNF115, FPR2, NPHP3, TFG, SGK223, C14orf64, HBG1, VWC2, C22orf23, GREB1L** |
| 9 | **UFSP2, TLR9, LOC100134259, CCND1, REG3G, TMPRSS11F, VPREB3, ZNF516, KIAA1383, LAMB2, IAPP, TRIM72, EI24, MED8, HYOU1, AGAP1, SCRT1, TM4SF18, FAM186B, CCL23, C4orf3, CLDN14, SNAP23, ASB17, CDC20B, EIF5AL1, CCL1, CENPBD1** |
| 8 | **NCRNA00171, DERL2, NFE2L3, PRKG2, COL19A1, MFSD2A, ZNF416, ZNF235, NGLY1, CSF2, GLP1R, F2RL1, CTSG, GANC, PLEKHA9, OR2L13, C20orf7, ZNF225, KEL, IL31RA, CROCCL2, DMKN, RTP3** |
| 7 | HERC1, CCDC79, PABPC4L, B3GALT1, LOC339535, LOC100289341, RBMXL2, ZNF222, KCNE1L, SLC26A8, CALCR, ZIC4, TAS2R41, ZFP161, CAPZA1, WNT16, ASH1L, HAUS1 |
| 6 | FOXP3, PCLO, OASL, FRMPD2, AK7, MAGEL2, KCNV1, H1F0, SULF1, CYP4F11, METTL10, EN2, LGI1, MTX1, ZBED2, SAA1, NOBOX, PTX3, TMEM57, C6orf191, EGR1, CMA1, FAM3B, NR5A1, LOC100130932 |
| 5 | TDRG1, ARPC3, HP, HHIPL2, ZNF773, ZNF815, C19orf22, ZZEF1, ZNF79, NKX2-2, SFRP4, RPIA, HIST1H3C, C14orf102, FOXD4, GDF10, COL9A1, LOC728554, DBX2, ZNF2, CD46, ZC3H12C, PSG11, ZNF23, LEPREL1, ALOX12B, PAGE5, KIAA1211, FLJ42875, DUXA, NKX2-3, CNIH4, CYP24A1, ZNF577, RIC8B, ZNF830, C6orf118, SNHG8, CIB4, TRIM45, MUC20, EIF3M |
| 4 | SLC10A6, SRPRB, C7orf42, USP27X, USP39, CHRNB4, DMRTC2, EXT1, PDIA2, DGUOK, SRP54, HOXA7, TRAK2, OSCAR, CDK2AP2, IL11, C1orf158, NEDD9, PHF7, PCDHA12, ZP4, HPR, SRD5A2, RNLS, TFAP2A, OTX1, CTAGE4 |
| 3 | TNNT1, R3HDM2, FOXM1, ACP2, KIAA1147, HSD11B1, ANGPT4, KLF13, SOX21, OR12D3, VAV3, EHHADH, CACNG1, LPIN2, HSPA1L, OBFC1, AMBP, PCCB, SNORA41, SPINK1, NR3C2, FZD9, C8orf84, PRDM8, DSC2, CELF4, BOD1L, SLC22A23, COL8A2, CTAGE9, CLEC3B, SULT1B1, FKBP1A, ARL11, LOC100130581, IQCH, LOC100268168, FGFR3, PABPC1L2A, TMEM54, ZCCHC14, DGAT2, CYP2A13, VASH2, ZNF555, AGAP8, GCNT3 |
| 2 | MOV10, C13orf34, C10orf113, SEMA3G, C1orf194, NPIPL3, NIPSNAP3B, INHBC, MAML2, CTHRC1, CXorf41, COL29A1, FLRT3, GPR112, GRAMD1B, C13orf1, NRXN2, KIF3B, KLF4, MUC7, EIF5B, ZNF629, CELA2A, SPZ1, ZNF440, TIMELESS, LOC339524, MGAT5B, RPSA, ADAMTS8, CLCN7, MTERFD2, CHML, SRP19, TGM4, HIST1H3I, SIK3, TRPC2, PPM1D, SIT1, SLC39A8, GOLT1A, SOX9, CDNF, CSAG3, ZNF274, FAM58B, ABCC11, IGSF11, SPACA5, PARP12, CTSF, KLF2, CD81, CD177, FAM89A, C12orf43, LOC441869, PRG3, MAST4, C9, C1orf68, C18orf8, CCDC134, NKX2-4, PCDHA13, TMEM158, ATP8A1, RPN2, FCN2, LONRF2, GPR56, TOB1, ATP4A, GAGE10, ASIP, GART, FAM9B, CHRAC1, FAM99A, OXCT2, GALR1, GPR45, ZNF699, PPP1R1A, HOXA13, RANGAP1, SMC5, SEC61A1, MBOAT7, WIPF3, C3orf1, WDR24, PTPRZ1, FGF5, OR4F5, ZIC1, SKA1, IL2RG, GZMA, TTC39B, KCTD14, TGFB3, CARHSP1, CDC14C, LEAP2, TMEM97, PSMD7, NCKIPSD, AJAP1, KISS1, CRMP1, LOC100128023, CXorf48, KLHL11, CDH11, RAB40B, ANKRD17, LOC653113, WSB2, HOXA1, AP2M1, ELOVL1, ZNF20, DAB1, MTCP1NB, SPIN4, LOC92973 |
| 1 | AURKB, METTL14, C21orf96, SNF8, TCEB1, CCDC91, ST7OT1, ZNF415, GRIK3, AUTS2, ADH4, LOC100132247, MGAT4B, MAGEE1, MRPL48, ADM, C17orf39, RPP40, NOMO3, TRIM42, DMRTC1, CXorf38, KANK4, UPK1A, TGM6, GNB5, NADK, IFIT2, STK32C, RABGGTA, KRTAP3-3, ADCYAP1, EPS15L1, C1orf146, HM13, ZNF593, CPM, ZNF583, TP53BP2, COMMD5, CCT2, ZRSR2, FGF12, APOBEC3A, DDHD1, DECR2, TMEM45A, TIPIN, OAZ3, EIF3K, HMBOX1, SMARCD1, SCARNA15, VSTM2L, NPVF, NDRG3, C14orf48, PITX2, ABCC12, LRP1, PSG1, C20orf24, TSHZ1, C20orf107, FAM54A, TNFRSF19, MNX1, ADCY9, C22orf27, TLR10, C7orf61, CSTB, NMI, JAKMIP3, FOXC2, PEMT, SP8, PNMA5, RPSAP52, EEF1G, TMEM41A, RASEF, P2RY2, CCDC27, ZNF689, C10orf84, VPS37B, NLRP14, FIP1L1, BMI1, LIPH, FABP7, ZNF648, APLNR, ANKRD10, TDRD6, AGER, SLC18A2, GTPBP2, LOC642846, ZNF709, NKX2-8, LOC100272217, LAGE3, MUC13, KRTAP10-6, KRT222, PIGH, CHEK2, WDR38, C4orf7, RASA4, TECPR1, FOSB, HCG11, KHK, TNFRSF10D, PCDHB8, SPO11, RWDD1, ZDHHC18, CD96, CYLC2, CCDC28B, NFYB, NRSN1, FREM2, ZNF836, CCNY, CRHBP, CCL19, NDUFB4, C6orf58, PSMB2, C12orf32, ADAMTS3, GCLC, ZPBP2, PTGR2, MAP2K5, ZFP82, ANGEL1, IL17D, LOC441294, IFNA10, MYCL1, KHDRBS2, OR8I2, CDRT15P, FBXW2, CH25H, MICALL2, TPPP, DNAJB11, GYLTL1B, C1R, AGAP11, SNX10, ACOT8, COMT, HFE2, C9orf68, TTC39A, ARID1B, YTHDC1, HOXC10, ALPK3, BEX1, SLC6A20, FLJ43663, STMN1, LMX1A, SEMA4F, KIAA0368, SSB, DPAGT1, OR2AT4, AQP12A, VWA5B2, EPC2, ZNF780B, TCP11, STK38, ECEL1, ?\|100134869, SGSM1, CIB1, CILP2, SPRR2D, FAM114A1, PADI2, C15orf53, PDGFRL, LOC283867, CHKB-CPT1B, PARN, NEIL1, SLC23A2, IL6, MYBPC2, LOC221122, PRAME, BTN3A3, PDIA3, MIDN, PTRH2, LOC145474, PRKCDBP, KCNJ13, ZNF530, SUSD1, FGFBP2, VILL, LOC100127888, COX4I2, SLC35B2, LOC441046, BCAS1, OR10G3, TCF7L2, C1orf83, SPRR2G, CES8, PRAMEF6, TPD52, PNLIP, KRTAP13-4, PDLIM4, LOC283050, PPP1CB, ENTHD1, OST4, LAMB3, PPP4C, IQSEC2, TAF3, PIK3R3, SETDB1, PFN1, TUBB8, PDPK1, MKI67, FGF7, GAB1, PLA2G5, C12orf72, ZNF324, TMEFF1, C13orf35, C10orf81, SLC24A3, GJB4, TOP1P2, LOC440905, MMP3, EFCAB10, PNPLA2, ASB1, SLFN11, TXNL4B, CCDC158, ZBTB16, GSX2, HSP90AB2P, FUCA2, MAGI1, GTSF1, CCNG2, TIRAP, CXCR3, SCARF2, NBPF1, RARA, CILP, KIAA0232, TMEM195, FAM179B, TRAFD1, C2orf55, HSPA5, IRF7, CDK6, AKNAD1, GOLGA6L5, BMP8A, TAX1BP3, KCNJ4, BCAR4, C19orf30, ZNF155, PTPRB, CEP55, ADAM8, TOR3A, AGPAT9, PPP1R1B, PYGO2, LOC100126784, SLC7A7, SLC25A22, PSMB9, C2orf40, NKX2-1, F10, ZNF835, SLC22A1, RNF41, LOC728819, TAS2R9, OR4F6, OR5F1, C2orf70 |

**Table S3.** Gene selection result of joint statistical measures, following 30 genes were removed during this process.

| reason | genes |
| --- | --- |
| IV<0.1 | LOC100134259, PRKG2, COL19A1, KIAA1383, CSF2, TRIM72, NODAL, EI24, TNMD, SCRT1, ZNF496, TM4SF18, TRPV5, FAM186B, CCL23, GANC, FAM167B, CHCHD2, C1orf97, ASB17, CRHR2, CBFA2T3, JPH2, FPR2, NPHP3, SGK223, VWC2, GREB1L, OR2L13, CCL1 |

**Table** **S4.** Functional roles of 23 hub genes with selected times ≥ 8.

| No. | Gene Symbol | Full Name or Aliases | Function |
| --- | --- | --- | --- |
| 1 | UFSP2 | UFM1 Specific Peptidase 2 | It encodes a highly conserved cysteine protease. |
| 2 | GPR68 | G Protein-Coupled Receptor 68 | A metastasis suppressor gene in prostate cancer. |
| 3 | RXRA | Retinoid X Receptor Alpha | It encodes nuclear receptors that mediate the biological effects of retinoids. |
| 4 | CACNA1D | Calcium Voltage-Gated Channel Subunit Alpha1 D | Associated with calcium-dependent processes |
| 5 | LOC285733 | - | A long non-coding RNA. Overexpression in urothelial carcinoma of bladder. |
| 6 | CASP9 | Caspase 9 | A member of the cysteine-aspartic acid protease (caspase) family. Involved in the activation cascade of caspases responsible for apoptosis execution. |
| 7 | PLA2G2A | Phospholipase A2 Group IIA | Associated with the colorectal cancer. |
| 8 | ATG13 | Autophagy Related 13 | An autophagy factor. Involved in autophagosome formation and mitophagy. |
| 9 | F2RL1 | F2R Like Trypsin Receptor 1 | Receptor for trypsin and trypsin-like enzymes coupled to G proteins. It is mediated through the activation of several signaling pathways. |
| 10 | GLP1R | Glucagon Like Peptide 1 Receptor | Involved in stimulating glucose-induced insulin secretion. |
| 11 | CTSG | Cathepsin G | May associated with the killing and digestion of engulfed pathogens, and in connective tissue remodeling at sites of inflammation. |
| 12 | KEL | Kell Metallo-Endopeptidase | It encodes a type II transmembrane glycoprotein. |
| 13 | HBG1 | Hemoglobin SubunitGamma 1 | Its expressed protein together with two αchains constitute fetal hemoglobin. |
| 14 | C22orf23 | Chromosome 22 Open Reading Frame 23 | - |
| 15 | LOC100132354 | LINC01512 | A non-coding RNA. May associated with lung adenocarcinoma. |
| 16 | HUS1B | HUS1 Checkpoint Clamp Component B | It participates in cell cycle arrest when DNA damage occurs. |
| 17 | STH | Saitohin | Associated with Ascaridiasis and Trichuriasis. |
| 18 | RNF115 | Ring Finger Protein 115 | Related in inner immunity. |
| 19 | CDC20B | Cell Division Cycle 20B | May related in the response of DNA damage. |
| 20 | MRGPRX2 | MAS Related GPR Family Member X2 | Associated with the G protein-coupled receptor activity. |
| 21 | CYCSP52 | Cytochrome C, Somatic Pseudogene 52 | A Pseudogene. It is involved in the antisense RNA class. |
| 22 | CENPBD1 | CENPB DNA-Binding Domain Containing 1 | A DNA-binding protein and it is derived from transposases of the pogo DNA transposon family. |
| 23 | IL31RA | Interleukin 31 Receptor A | Involved in IL-31 pathway. May functions in skin immunity. |

**Table S5**. The performance of machine learning-based models using the value of FPKM and RSEM respectively

| Data Type | Algorithms | Methods | Performance Measures | | | | |
| --- | --- | --- | --- | --- | --- | --- | --- |
|  |  |  | Sensitivity | Specificity | Accuracy(%) | MCC | AUC |
| FPKM | Discretization  + LR | Testing | 0.583 | 0.861 | 75.00 | 0.468 | **0.829** |
| RSEM | Discretization  + LR | Testing | 0.756 | 0.767 | 77.87 | 0.554 | 0.860 |

Reference:

[1] Pedregosa, Fabian, et al. "Scikit-learn: Machine learning in Python." Journal of machine learning research 12.Oct (2011): 2825-2830.

[2] Witten, Ian H., and Eibe Frank. "Data mining: practical machine learning tools and techniques with Java implementations." Acm Sigmod Record 31.1 (2002): 76-77.

[3] Bhalla, Sherry, et al. "Gene expression-based biomarkers for discriminating early and late stage of clear cell renal cancer." Scientific reports 7 (2017): 44997.
